# Acute appendicitis in a patient with sub-hepatic, sub-serosal, and retroperitoneal location. An intraoperative management challenge

**DOI:** 10.1016/j.ijscr.2024.110540

**Published:** 2024-10-29

**Authors:** Dagmawi Anteneh Teferi, Shanko Gebru, Alexander Tewodros Kassa, Helina Amare Abebe, Solomon Fekadu Yehualawork, Wubhareg Anteneh Teferi

**Affiliations:** a Department of Surgery, St. Paul's Hospital Millennium Medical College, Addis Ababa, Ethiopia; b Butajira General Hospital, Butajira, Central Region, Ethiopia

**Keywords:** Acute abdomen, Appendicitis, Subhepatic appendix, Retroperitoneal appendix, Subserosal appendix, Appendectomy

## Abstract

**Introduction:**

Appendicitis in patients with a sub-hepatic and retroperitoneal position is rare, often leading to delayed diagnosis and management due to its atypical presentation. A high index of clinical suspicion and the use of imaging modalities can improve the outcomes of patients with sub-hepatic appendicitis.

**Case presentation:**

A 20-year-old male presented with 36 h duration of right-sided abdominal pain, accompanied by nausea, vomiting, anorexia, and fever. He exhibited tachycardia and right lower quadrant abdominal tenderness. Laboratory tests revealed leukocytosis with a left shift and ultrasound showed simple appendicitis. An open appendectomy revealed a retroperitoneal, sub-hepatic, and sub-serosal inflamed appendix. The patient's postoperative course was uneventful.

**Clinical discussion:**

Sub-hepatic position of the appendix is rare accounting for 0.08 % of all cases of acute appendicitis. It is associated with mid-gut mal-rotation or arrested cecal descent during embryogenesis. Patients with sub-hepatic appendicitis usually have atypical presentation mimicking hepatobiliary pathologies which will lead to a delayed diagnosis and management. The standard management of sub-hepatic, retroperitoneal, and sub-serosal appendicitis relies on a laparoscopic approach however in case of difficulty and resource limitation, open appendectomy is the ultimate option.

**Conclusion:**

Sub-hepatic retroperitoneal and sub-serosal appendicitis, though rare, should be included in the differential diagnosis for patients with atypical abdominal pain. A high index of clinical suspicion, use of imaging modalities, and meticulous dissection with adequate exposure are crucial for a successful outcome.

## Highlights

• Sub-hepatic, retroperitoneal, sub-serosal appendicitis is a rare occurrence with diagnostic and management challenges
• Imaging modalities have a great role in guiding the diagnosis and surgical interventions of sub-hepatic appendicitis
• Colonic mobilization along with a meticulous dissection is crucial in managing retroperitoneal and sub-serosal appendicitis

## 1 Introduction

**Fig. 1:**
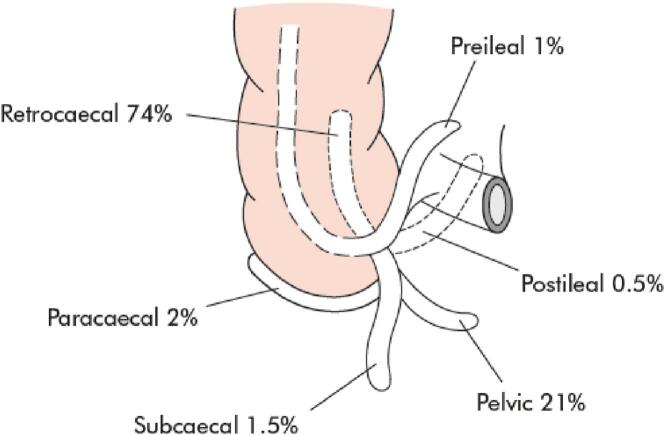
Different anatomic positions of the appendix with their relative prevalence [7].

Appendicitis is an inflammation of the vermiform appendix, a mid-gut intraperitoneal structure typically located in the right lower quadrant of the abdomen, but it can have various anatomical positions. The most common position is retro-caecal (74 %), followed by pelvic (21 %). Other positions include sub-caecal (1.5 %), pre-ileal (1 %), and post-ileal (0.5 %) [1] as shown in Fig. 1. A sub-hepatic appendix results from the failure of the appendix and cecum to descend during embryogenesis, accounting for 0.08 % of acute appendicitis cases and presenting atypically [2].

Acute appendicitis is a common surgical emergency, representing 4–8 % of all emergency department visits and 38.9–46.9 % of acute abdomen cases requiring emergency surgical intervention in adults [[3], [4], [5]]. Diagnosing acute appendicitis typically relies on clinical symptoms, signs, and radiologic evidence. However, anatomical variations can lead to atypical presentations, delayed diagnosis, and late surgical intervention; resulting in the development of complications [6].

This report presents a rare case of sub-hepatic appendicitis and discusses the challenges encountered in its surgical management. We present our case in line with the SCARE criteria [8].

## 2 Case presentation

A 20-year-old male presented with initial periumbilical crampy abdominal pain, which shifted to the right lower quadrant and flank area of 36 h duration. The patient had a low-grade fever, nausea, one episode of vomiting, and loss of appetite. There was no history of diarrhea, abdominal distention, or urinary symptoms. The patient had no chronic medical conditions or prior surgical history.

**Fig. 2:**
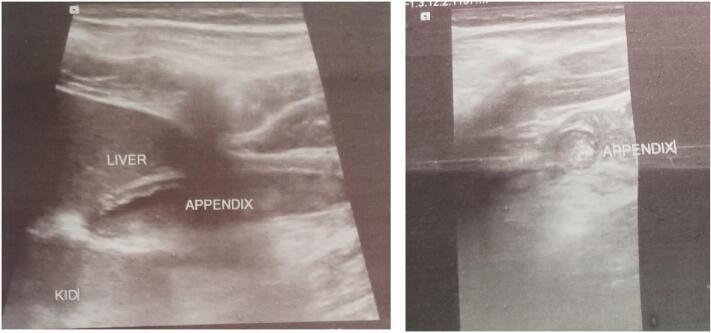
Abdominopelvic ultrasound showing an enlarged non-compressible appendix measuring 9 mm with edematous and thick wall, and fat stranding.

On examination, the patient was in pain, with a blood pressure of 110/74 mmHg, pulse rate of 104 beats per minute, respiratory rate of 18 breaths per minute, temperature of 37.8 °C, and random blood sugar of 98 mg/dl. Abdominal examination revealed direct and rebound tenderness over the right lower and upper quadrant. Laboratory investigations showed leukocytosis of 13.3 × 10^3^/mm^3^ with a left shift of 82.6 %. Other tests, including urine analysis and renal function tests, were unremarkable. Abdominal ultrasound revealed a non-compressible retro-caecal appendix measuring 9 mm with a thick, edematous wall, mesenteric fat stranding, and tenderness on probe compression (Fig. 2).

**Fig. 3:**
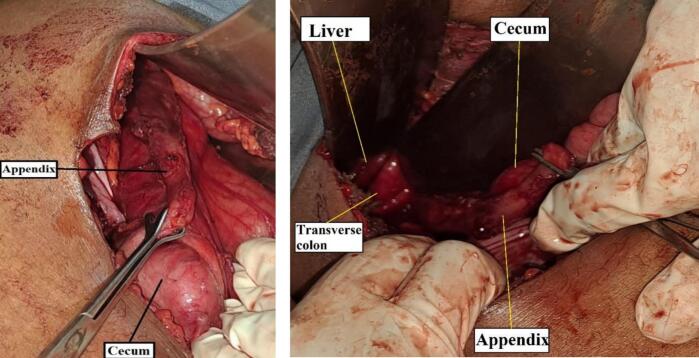
Intraoperative picture of retroperitoneal, sub-hepatic sub-serosal appendix depicted in both figures along with cecum, transverse colon, right para-colic gutter, and liver.

The patient Alvarado's score is 9/10 and the ultrasound is suggestive of acute appendicitis. The patient was kept nil per os (NPO), resuscitated with 1000 ml of normal saline, and started on intravenous ceftriaxone, metronidazole, and analgesics. After obtaining written informed consent for surgery, the patient was taken to the operating theatre. The abdomen was approached through a Rocky-Davis incision. However, it was difficult to access the appendix and the incision was extended laterally and vertically. Despite having adequate exposure to access the right side of the intraperitoneal organs, the distal segment of the appendix was not accessible. Subsequently, the retroperitoneum was opened on the right side through an incision made over the line of toldt where the distal part of the appendix was identified retroperitoneally extending cranially towards the liver. The appendix was sub-serosal in its proximal segment with a healthy base and there was phlegmon at its tip and adhesion with the colon but no collection. The appendix was gently dissected from the cecum, ascending colon, and hepatic flexure of the transverse colon, and appendectomy was done (Fig. 3).

The patient had a smooth postoperative course and was discharged on the third postoperative day without any complications. Subsequently, the patient was seen on postoperative days 7 and 30 and had no any complication.

## 3 Discussion

The sub-hepatic position of the appendix accounts for 0.08 % of all cases of acute appendicitis [2]. It occurs in association with mid-gut mal-rotation or arrested cecal descent during embryogenesis. Arrested cecal descent occurs where the caecum lies in the sub-hepatic position but does not descend to the right iliac fossa. The appendix is an intraperitoneal organ originating from mid-gut, however, in 7 % of the cases it was found to have a retroperitoneal position [9].

Patients with sub-hepatic appendicitis usually have atypical presentation usually mimicking acute cholecystitis, liver abscess, acute pancreatitis, and intestinal perforation/obstruction. This will lead to a delayed diagnosis and management that will eventually complicate with peritonitis [1]. This is not the case in our patient where he presented with classical symptoms and signs of acute appendicitis.

High index of clinical suspicion and thorough imaging evaluation by ultrasound or CT scan, are essential for accurate diagnosis [1]. In this case, ultrasound identified the inflamed appendix despite not reporting its atypical location.

The standard surgical management of acute appendicitis is laparoscopic appendectomy irrespective of the position of the appendix and the development of local complications [10]. However, due to the absence of expertise and gadgets, only open appendectomy is being done in our setup.

Access to the appendix during open appendectomy in case of uncomplicated appendicitis is through Gridiron, Lanz, or Rocky Davis incision. Lanz and Rocky Davis incisions have better cosmetic outcomes as compared to the oblique Gridiron incision as it is made along the langer's line of the skin tension and can be covered with a bikini despite the risk of injury to ilio-inguinal and ilio-hypogastric nerves. Alternatively, based on preoperative imaging, an incision can be made using the point of maximal tenderness or appendiceal location if we anticipate localized complications or intraoperative difficulty. Otherwise, if the patient has evidence of peritonitis, midline vertical incisions are recommended [7,9].

The standard open appendectomy approach may need modification in case of sub-hepatic appendicitis for better exposure and access. The routine Rocky-Davis incision for uncomplicated acute appendicitis is not favorable for extension in the case of sub-hepatic appendicitis rather an oblique right lower quadrant or vertical midline incision is preferred [11,12]. In our case, we extended the incision laterally and additional vertical extension was made which is a bit cosmetically unsightly and could have been avoided if the sub-hepatic position of the appendix was known preoperatively.

Mobilizing the cecum and ascending colon is recommended to remove the inflamed appendix in case of retroperitoneal location [12]. In our case, the cecum and ascending colon were mobilized to access and remove the appendix. The presence of inflammatory changes along with the sub-serosal location of the appendix makes the colon prone to injury and perforation as the tissue is fragile and the appendix is buried within the layer of the colon. This warrants a meticulous dissection to separate the appendix from the colon. The occurrence of these complications might change the subsequent course of surgical management and follow-up significantly.

## 4 Conclusion

This case emphasizes the importance of considering anatomical variations in diagnosing and managing acute appendicitis. Despite its rare occurrence, sub-hepatic and retroperitoneal appendicitis should be considered as a differential diagnosis in patients with atypical abdominal pain. High clinical suspicion, appropriate imaging, and surgical expertise are critical for effective management.

Availing expertise and gadgets for laparoscopic appendectomy would have avoided unnecessary incision extensions and complications.

## Ethical approval

Ethical approval is deemed unnecessary by the hospital ethics committee as this is a rare case faced during clinical practice and doesn't involve experiments in humans or animals.

## Consent for publication

Written informed consent was obtained from the patient for publication and use of images. The written consent is available for review by the Editor-in-Chief of this journal upon inquiry.

## Guarantor

Dagmawi Anteneh Teferi.

## Research registration number

None.

## Funding

There is no source of funding for this study.

## Author contribution

**Dagmawi Anteneh Teferi**: Study conceptualization and design, original draft write-up, data curation, paper review & editing, and patient management.

**Shanko Gebru**: Study conceptualization and design, original draft write-up, data curation, paper review & editing, and patient management.

**Alexander Tewodros Kassa**: Study conceptualization and design, original draft write-up, data curation, paper review & editing, and patient management.

**Helina Amare Abebe**: Study conceptualization and design, original draft write-up, data curation, paper review & editing, and patient management.

**Solomon Fekadu Yehualawork**: Study conceptualization and design, original draft write-up, data curation, paper review & editing, and patient management.

**Wubhareg Anteneh Teferi**: Study conceptualization and design, original draft write-up, data curation, paper review & editing.

## Conflict of interest statement

The authors declare no conflict of interest.
